# Publish or Perish mantra in the medical field: A systematic review of the reasons, consequences and remedies

**DOI:** 10.12669/pjms.326.10490

**Published:** 2016

**Authors:** Salman Y. Guraya, Robert I. Norman, Khalid I. Khoshhal, Shaista Salman Guraya, Antonello Forgione

**Affiliations:** 1 Prof. Salman Y. Guraya, FRCS, Masters MedEd(Dundee). Consultant Colorectal Surgeon, College of Medicine, Taibah University, Almadinah Almunawwarah, Saudi Arabia; 2 Dr. Robert I. Norman, B.Sc, Ph.D. Academic Director, College of Medicine, Biological Sciences and Physiology, University of Leicester, United Kingdom; 3 Prof. Khalid I. Khoshhal, FRCS. Consultant Pediatric and Orthopedic Surgeon Vice Rector, Research and Graduate Studies, Taibah University, Almadinah Almunawwarah, Saudi Arabia; 4 Dr. Shaista Salman Guraya, Ph.D. Assistant Professor of Radiology, College of Medicine, Taibah University, Almadinah Almunawwarah, Saudi Arabia; 5 Dr. Antonello Forgione, MD. Consultant Colorectal Surgeon, Department of General and Emergency Surgery, NiguardaCàGranda Hospital, Director, AIMS Advanced International Mini-invasive Surgery Academy, Milan, Italy

**Keywords:** Academic publications, Pressure to publish, Plagiarism, Retraction, Research ethics, Medical field

## Abstract

**Objectives:**

Generally, academic promotions, job retention, job mobility, and professional development of a medical faculty members are judged primarily by the growth in publication outputs. Universities and research institutions are more likely to recruit and promote those academics carrying voluminous résumés with larger number of published articles. This review elaborates the causes and consequences of the pressure to publish and the ways and means to cope with this paradigm.

**Methods:**

In 2015, database of Abstracts of Reviews of Effects, LISTA (EBSCO), Medline and Oxford University Library were searched for the English language full-text articles published during 2000-2015, by using MeSH terms “pressure to publish”, “urge to publish”, “research ethics”, “plagiarism”, “article retraction”, “medical field”. This search was further refined by selecting the articles in terms of relevancy and contents.

**Results:**

This research showed that some universities offer generous grants to researchers with a high h-index and with more publications in elite journals, which promise an enhanced prospect of citations and elevation in the scientific rankings of the funding institutions. This generates an involuntary obsession to publish with the primary intention to obtain promotions, high scientific rankings, and improved job security. This compelling pressure to publish results in widespread publication of non-significant research with a high index of plagiarism that eventually leads to an increased frequency of retractions.

**Conclusion:**

Research centers and academic institutions have an obligation to train their academics in sound scientific writing and to apprise them of the publication ethics and the grave consequences of plagiarism and research misconduct.

## INTRODUCTION

Failure to publish novel scientific work by a researcher deprives the world of information and evidence-based innovations. Scientific publications are the triggers that motivate other scientists.^1^ Published knowledge stimulates more research; hypotheses are modified, rebutted or endorsed, and new paradigms are introduced at the expense of the old ones. Published literature is a legacy to science. Writing research for publication is the final frontier in the research endeavor that can be challenging and sometimes disappointing due to rejection by elite journals and negative peer review decisions. However, these barriers should not deter the scientist from publishing their research findings. Publication is a very effective communication channel for the scientists to share their work, ideas, difficulties, and achievements.

In addition to scientific communication, publication has become vitally important for the physicians for a number of other reasons such as professional development,^2^ compulsory regulatory obligations, requirements for promotion, monetary incentives, and the wish to progress in the scientific community.^3^,^4^ There is a compelling urge to publish research across the globe in general and in the developing countries in particular, which accounts for more than two-thirds of the world population.^5^,^6^ The abundance of clinical data in developing countries provides great opportunities for research and publication, however, a lack of research training and expertise in terms of designing a research protocol design, rigorous methods for quality data collection and storage, relevant statistical analyses, and scholarly writing are major barriers to publishing research in reputed journals.^7^

This review explores the factors that influence the academic physicians to hurriedly publish their research in response to the pressure of their promotions, survival at the workplaces, potential financial perks and the growth of their research portfolio. The outcomes of this urge to publish are also outlined and some plausible remedies are suggested.

### Research design

**Fig.1:**
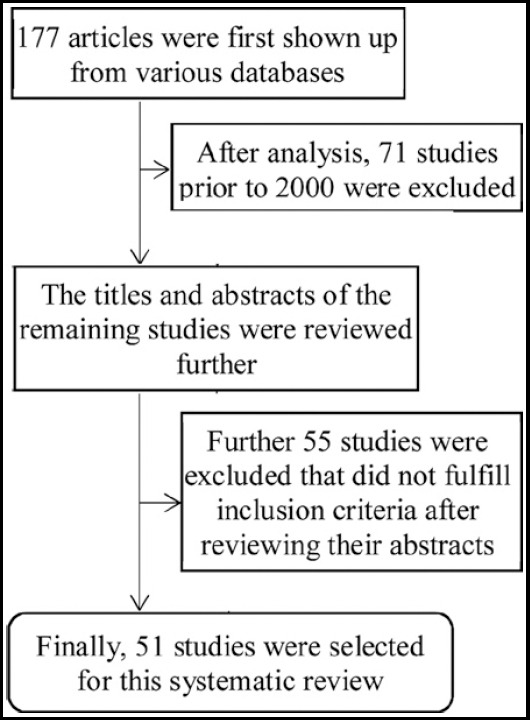
Schematic presentation of selection of studies in this systematic review about the publish or perish mantra in the medical field.

The online search mode of EndNote X 5 was used to review the published literature of the English-language articles in Medline, ISI web of knowledge, ScienceDirect, the Cochrane Database of evidence-based reviews, Database of Abstracts of Reviews of Effects, LISTA (EBSCO), and Oxford University Library during the period 2000-2015. MeSH terms “pressure to publish”, “urge to publish”, “research ethics”, “plagiarism”, “article retraction”, “medical field” were used to search for the selected articles. This search retrieved 177 articles that were further refined in terms of the relevancy of topic and the application and validity. Finally, a total of 51 articles were selected for this review. The process of final selection of studies used in this systematic review is shown as a flow chart in Fig.1.

This systematic review generated diverse themes about the reasons and consequences of publish or perish and a framework of remedies to cope with this unethical practice have been elaborated in the following section

### A. Reasons for pressure to publish

### 1. Recruitment criteria

The scientific output of departments, faculties, programs and ultimately the university contribute to the overall scientific stature of that particular institution.^6^,^8^ This scientific ranking helps outside stakeholders assess the quality of research.^9^ Thus the benchmark for the evaluation of institutional ranking is often the quantity rather than quality. The pressure to publish mantra becomes relevant even before graduation when residents apply for training programs in their specialties. Interestingly, an analysis of published articles in the *Journal of Pediatric Surgery* over the three decades to 2010 showed a striking increase in the number of authors per article. This was interpreted to suggest greater complexity in ways of working along with a pressure to develop publication-rich résumés.^10^ A compelling reason behind this increase in the number of authors per article is the enormous pressure to publish.^11^ A PhD candidate or a postdoc working under the supervision of an eminent scholar is perceived to have a more impressive resume than others.^12^ However, this does not necessarily mean that the researcher is professionally more active and competent. It could simply mean that he or she was in the right place at the right time.

An academician’s professional stature is often measured by the number of peer-reviewed articles, by the impact factor of the journals publishing the research, and by the number of citations to the work.^13^ These key performance indicators are carefully considered by institutional recruitment committees and the applicants with a longer list of publications carry more chances of success.

### 2. Scientific rankings of institutions

In the recent past, a polite reminder to publish by institutions has been transformed into a “publish or perish” paradigm of the academic professional life.^14^ Research output in terms of publication numbers are used for the measurement of institutional performance which in turn plays a pivotal role in the scientific rankings of institutions in the region and worldwide.^15^ Some institutions recruit and generously pay eminent writers in order to raise their rankings and scientific stature. The purely monetary incentives to attract prominent researchers with their promising bibliographies can assist universities to climb several hundred places in international rankings regardless of whether the work was done at their institution.^16^ This institutional emphasis to increase the research output by academia may jeopardize other equally important academic activities such as clinical and educational duties.^17^

### 3. Academic promotions and publications

In response to the highly competitive environment within academic institutions, researchers are tempted to cut corners for publishing articles in an attempt to fulfill the eligibility for promotions and to win the candidacy for extra-ordinary academic achievements awards.^18^ Academia’s performance indicators of publications, citations and impact factors pressurizes the faculty to stretch unethically beyond the boundaries of ethics in medical research.^19^ At the same time, this competition has led to a sharp rise in the prevalence of ‘guest’ and ‘ghost’ authors.^20^ Ghost authors refer to those who never contributed to the research or publications, and get the authorship through their relationships or posts. Guest authors are those with a high *h*-index, added by novice writers merely to amplify the chances of publication in high impact journals. Both of these categories belong to research misconduct.^21^

### 4. Perks from pharmaceutical companies

Enormous funding for clinical trials, publishing and archiving enterprises is provided by various multi-national pharmaceutical companies. These pharmaceutical agencies support individual researchers as well as institutions, for their marketing and business. Frequently, these agencies recruit professional writers and eminent scientists to draft reports of clinical trials. These efforts are heavily rewarded and create undue pressure on the writers to publish the results in order to receive the incentives and rewards.^22^ Under this pressure to publish, the findings of the research sometimes clash with conflicts of interests and the safety of patients may also be jeopardized.^23^

### 5. Nontenured faculty

The tenure system poses a potential threat to the nontenured faculty and those holding an honorary adjunct academic title.^24^ Their appointments can be terminated without a prior notice, thus placing them in a vulnerable situation. To circumvent this potential threat, ontenured faculty, in an attempt to prove their productivity, can be tempted to publish heavily in an unethical and non-scientific manner.

### 6. Institutional grants and funding to writers publishing in prestigious periodicals

Authors publishing in high-ranked journals attract more citations, secure a high *h*-index and, in turn, receive lucrative grants from the research centers and governmental funding agencies. Organizations target top researchers with very high *h*-indices, and challenge them to publish in reputed journals to boost the institution’s scientific rankings. Ultimately, high rank journals can fall prey to unethical publication and plagiarized work, which consequently end up with retractions.^25^ Retraction is a mechanism of correcting the literature and informing readers about publications with seriously flawed or unreliable and erroneous data.^26^ Unreliable data may result from intentional manipulations of the data or previously published information. *Proceedings of the National Academy of Sciences* notified eight and *Science* announced five retractions in 2011.^27^ Steen RG evaluated all 742 English-language Pubmed Central indexed retracted articles during 2000 to 2010 and showed that 73.5% of articles were retracted due to errors and 26.6% articles due to falsification and fabrication of the data.^28^ The author also reported a sharp rise in annual retractions over the last decade [r=0.87; *P*< 0.001]. The pressure to publish has been coined as the main driving force in compelling the authors to commit scientific fraud and research misconduct.^29^

### B. Consequences of pressure to publish

### 1. Article retractions

In order to accommodate the ever-increasing number of submissions to journals and publishers, an explosive growth of new publishers have sprung up globally, and the number of online and subscription-based journals has increased exponentially.^30^ At the same time, the pressure to publish at all costs has led to increasing episodes of research misconduct and inevitable retractions, which then questions the integrity of the current published literature.^31^ The world-renowned publishers like Nature, Science and Cell carrying very impressive impact factors have witnessed the highest rates of retractions.^32^ This rise in retractions is helping the growth of predatory journals and publishers as these dubious publishing portals promise quick publication for hard cash. Factors contributing to retractions include plagiarism, multiple simultaneous submissions and duplicate publications, research misconduct in the form of breach of copyrights and ethical guidelines for research and ghost authorships.^33^ A rigorous post-publication scrutiny by high-profile publishers has exerted a modest impact on retractions.^34^ Wager and Williams analyzed all available Medline retractions during the period 2005 to 2008 and one-in-three randomly selected retracted articles during the period 1988 to 2004.^35^ The reasons for retractions included intentional error or non-replicate findings [40%], issues of ethical and research misconduct [17%], and redundant publications [17%]. Similarly, a 20-fold increase in the number of retractions in journals indexed by the Science Citation Index Expanded has been reported.^36^ The majority of retractions has been found to be due to wrongly designed research or poorly crafted manuscripts resulting from the pressure to publish syndrome. Retractionwatch.com has recently pointed out authors orchestrating fake peer reviews by submitting false contact information for suggested reviewers, companies selling fake peer reviews to get articles published, and elite researchers publicly selling their writing skills on social media.^37^

### 2. Plagiarism

Plagiarism, a scientific theft, primarily stems from the urge to publish more and a lack of writing skills.^38^,^39^ The publication of various forms of plagiarism such as ‘self-plagiarism’, ‘redundant publication’, ‘duplicate publication’, or ‘salami publishing’, have increased sharply five-fold from 170 in 2000 to 820 in 2012.^40^ Plagiarism has become a global problem for the researcher community due to the easy access to Internet resources. Lack of ethical awareness, inadequate language skills, and inappropriate application of information [e.g. summarizing, paraphrasing and quotations], unfamiliarity with Western scholarly traditions, speedy inflation of one’s scientific ranking and pressure to publish are the most commonly cited reasons for plagiarism.^41^-^43^ A number of systems are commercially available that can detect plagiarism and can identify the plagiarized parts of an article. Examples of these available sites are Turnitin, iThenticate, eTBLAST, Copyscape and Viper. These sites are widely used by editorial teams when a publication is being reviewed, by authors before submitting an article for publication and by universities in order to evaluate the quality of student thesis and dissertations.^44^

### 3. Working atmosphere

The pressure to publish can drain some of the joy of practicing science.^45^ The most catastrophic effect is witnessed inside research labs, where the real professional competitive atmosphere among junior scientists is deteriorating. It insidiously undermines relations between colleagues. Junior scientists think they have no choice but to join the race for publications as the only way to survive and excel in their career.^46^

### C. Remedies for ‘publish or perish’

Literature has proposed a number of remedies to rectify the ‘pressure to publish’ continuum in the medical literature. Some important steps are described hereunder:

In 2005, the International Committee of Medical Journal Editors [ICMJE] introduced a policy requiring investigators to submit information about the protocol of a clinical trial in an official clinical trials registry before the start of patient recruitment.^29^ This policy would ensure that information about the design and conduct of clinical trials was publicly available.^47^ By enrolling with the clinical trials registry, researchers will have an official testimony with regard to the content and scientific validity of the research.

A proliferation of publications by faculty at the expense of other areas of academic performance such as innovative teaching strategies and one-on-one involvement with students undermines faculty output.^48^ Giving less weightage to the number of publications required for academic promotions and tenure decision-making will alleviate considerable faculty stress.

“If candidates were only allowed to submit articles for which they were principally responsible, the result would be more papers that reflect the contribution of a few key authors, which would allow more junior researchers to develop their own research initiatives rather than merely appearing in a laundry list of contributors”.^48^

Instead of rewarding the faculty with enormous publications, a suggested way would be to require the faculty members to submit an arbitrary number of papers that they consider to have produced significant impact in scientific literature.^49^ This would arrest the competition to publish articles for promotions and other publication-linked incentives.

Research and publishing need different construct of skills as scholarly writing is a systematic way that can make the presentation easily reproducible and understandable even for the novice.^50^,^51^ Writing for publication is a multi-stage process that involves writing an initial draft, revising and finalizing the manuscript, and then submission, revision, resubmission, and proofreading for the journal/publisher.

Medical writers should be encouraged to attend training workshops and seminars to enhance their writing and publishing skills.^52^ Understanding the ethical principles applicable to research and writing will deter researchers from cutting the corners for pharmaceutical companies and for their own personal benefits.

Scientific journals should carefully assess the accuracy of research data, authorship rights, authors’ contributions, conflict of interests, ethical approvals, and the funding agencies for research grants.

The Committee on Publications Ethics [COPE] strongly recommends that institutions should assign a focal person for dealing with research integrity and misconduct allegations.^21^ This exercise will discourage the wrong doers and help patronize genuine research output.

The research community should invest in mechanisms to assess research quality, rather than using metrics-based assessments of individual and institutional research outputs. This would initiate value judgments of the quality of research publications to drive up quality and discourage unscholarly practice.

## CONCLUSION

Original research performed in a systematic manner and published in a reputed journal is highly desirable for the exchange of scientific information and the contribution to developments in the field, however, the pressure to publish can clearly undermine the educational mission of institutions due to questionable research output. Academic promotions, incentives by the pharmaceutical companies, award of grants and funding, and the competition for the scientific rankings of individuals and institutions can contribute negatively to novel academic practice. Publications of non-significant or weak scientific work spoil the integrity and validity of the entire research process. An effective publish or perish paradigm should be based on the appraisal of the quality of scientific content rather than the quantity. Originality, relevance, excellence and integrity of published medical research can only be ensured by the combined efforts of institutions, writers, reviewers, editors and publishing working together to maintain and improve the quality of research outputs.

## References

[1] Clapham P (2005). Publish or perish. Bio Science.

[2] Tonges M (2000). Publishing as a career development tool: don’t forget to write. Seminars for Nurse Managers.

[3] Dixon N (2001). Writing for publication–a guide for new authors. Int J Quality Health Care.

[4] Plümper T, Radaelli C (2004). Publish or perish? Publications and citations of Italian political scientists in International Political Science Journals 1990-2002. J Euro Public Policy.

[5] Sengupta S, Shukla D, Ramulu P, Natarajan S, Biswas J (2014). Publish or perish: The art of scientific writing. Indian J Ophthalmol.

[6] Linton JD, Tierney R, Walsh ST (2011). Publish or perish: How are research and reputation related?. Serials Rev.

[7] Solagberu BA (2002). Literature search in medical publications. West African J Med.

[8] Cyrenne P, Grant H (2009). University decision making and prestige: An empirical study. Economics Educ Rev.

[9] Boulton G (2011). University rankings: Diversity, excellence and the European initiative. Procedia-Social and Behavioral Sciences.

[10] Pintér A (2013). Changing trends in authorship patterns in the JPS: Publish or perish. J Pediatr Surg.

[11] Neill US (2008). Publish or perish, but at what cost?. J Clin Invest.

[12] Brischoux F, Cook TR (2009). Juniors seek an end to the impact factor race. Bio Science.

[13] Low W-Y, Ng K-H (2011). International Collaboration in Journal Publishing Enhancing Quality and Visibility. Asia-Pacific J Public health.

[14] Parchomovsky G (2000). Publish or perish. Michigan Law Rev.

[15] McGrail MR, Rickard CM, Jones R (2006). Publish or perish: a systematic review of interventions to increase academic publication rates. High Educ Res Dev.

[16] Bhattacharjee Y (2011). Saudi universities offer cash in exchange for academic prestige. Science.

[17] Tijdink JK, Verbeke R, Smulders YM (2014). Publication Pressure and Scientific Misconduct in Medical Scientists. J Empir Res Hum Res Ethics.

[18] Casadevall A, Fang FC (2012). Reforming science: Methodological and cultural reforms. Infect Immun.

[19] Brice J, Bligh J (2005). Author misconduct: not just the editors’responsibility. Med Educ.

[20] Matias-Guiu J, Garcia-Ramos R (2011). Ghost-authors, improvement article communication, and medical publications. Neurología (English Edition).

[21] Wager E, Kleinert S (2012). Cooperation between research institutions and journals on research integrity cases: Guidance from the Committee on Publication Ethics (COPE). Maturitas.

[22] Salari P, Namazi H, Abdollahi M, Khansari F, Nikfar S, Larijani B (2013). Code of ethics for the national pharmaceutical system: Codifying and compilation. J Res Med Sci.

[23] Bayrami Z, Abdollahi M (2011). Observance of ethical codes in selecting supervisor by postgraduate students. J Med Ethics History Med.

[24] Zhou Y, Volkwein JF (2004). Examining the influences on faculty departure intentions: A comparison of tenured versus nontenured faculty at research universities using NSOPF-99. Res High Educ.

[25] Gasparyan AY, Ayvazyan L, Akazhanov NA, Kitas GD (2014). Self-correction in biomedical publications and the scientific impact. Croatian Med J.

[26] Wager E, Barbour V, Yentis S, Kleinert S (2009). Retractions: Guidance from the Committee on Publication Ethics (COPE). Maturitas.

[27] Fang FC, Casadevall A (2011). Retracted science and the retraction index. Infect Immun.

[28] Steen RG (2010). Retractions in the scientific literature: is the incidence of research fraud increasing?. J Med Ethics.

[29] Fang FC, Steen RG, Casadevall A (2012). Misconduct accounts for the majority of retracted scientific publications. Proceedings of the National Academy of Sciences.

[30] Abdollahi M, Gasparyan AY, Saeidnia S (2014). The urge to publish more and its consequences. DARU J Pharma Sci.

[31] Budd JM, Coble ZC, Anderson KM (2011). Retracted publications in biomedicine: Cause for concern.

[32] Jawaid SA (2016). Publish or Perish: Need to have another look?. Pak J Med Sc.

[33] Decullier E, Huot L, Samson G, Maisonneuve H (2013). Visibility of retractions: a cross-sectional one-year study. BMC research notes.

[34] Steen RG, Casadevall A, Fang FC (2013). Why has the number of scientific retractions increased?. PloS One.

[35] Wager E, Williams P (2011). Why and how do journals retract articles? An analysis of Medline retractions 1988–2008. J Med Ethics.

[36] Zimmer C (2012). A sharp rise in retractions prompts calls for reform.

[37] (2014). www.retractionwatch.com.

[38] Das N, Panjabi M (2011). Plagiarism: Why is it such a big issue for medical writers?. Perspect Clin Res.

[39] Steen RG (2011). Misinformation in the medical literature: what role do error and fraud play?. J Med Ethics.

[40] Martin BR (2013). Whither research integrity? Plagiarism, self-plagiarism and coercive citation in an age of research assessment. Res Policy.

[41] Colnerud G, Rosander M (2009). Academic dishonesty, ethical norms and learning. Assess Eval High Educ.

[42] Jackson PA (2006). Plagiarism instruction online: Assessing undergraduate students’ability to avoid plagiarism. Coll Res Libr.

[43] Sisti DA (2007). How do high school students justify internet plagiarism?. Ethics Behav.

[44] Tsiligianni IG, van der Molen T (2011). Plagiarism: Are There Practical Ways to Avoid It?. J Peri Anesth Nurs.

[45] Raff M, Johnson A, Walter P (2008). Painful publishing. Science-New York Then Washington.

[46] Cherubini P (2008). Impact factor fever. Science (New York, NY).

[47] Laine C, Horton R, DeAngelis CD, Drazen JM, Frizelle FA, Godlee F (2007). Clinical trial registration. BMJ.

[48] Liewehr FR (2005). Time to rethink publish or perish. Oral Surg Oral Med Oral Pathol Oral Radiol Endodontol.

[49] Hussey R (2007). The pretence of publishing: A beneficial conspiracy for academics. Account Educ.

[50] Moos DD (2011). Novice authors… What you need to know to make writing for publication smooth. J Peri Anesthesia Nurs.

[51] Hasse JM (2013). Developing the “Write” Skills for Publishing. Nutr Clin Pract.

[52] Alberts B, Hanson B, Kelner KL (2008). Reviewing peer review. Science.

